# Value of Transverse Groove on the Earlobe and Hair Growth on the Ear to Predict the Risk for Coronary Artery Disease and Its Severity among Iranian Population, in Tehran City

**DOI:** 10.31661/gmj.v9i0.1443

**Published:** 2020-05-22

**Authors:** Reza Arefi, Mohammad Hassan Namazi, Morteza Safi, Habiboulah Saadat, Hossein Vakili, Mehdi Pishgahi, Saeed Alipour Parsa

**Affiliations:** ^1^Research Committee of AJA University of Medical Science, Tehran, Iran; ^2^Shahid Beheshti University of Medical Sciences, Cardiovascular Research Center, Tehran, Iran

**Keywords:** Groove, Earlobe, Hair Growth, Coronary Artery Disease, Iranian Population

## Abstract

**Background:**

The use of phenotypic parameters along with other noninvasive diagnostic modality can lead to early diagnosis of coronary artery disease (CAD) and prevent its life-threatening outcome. Recently, the application of head and face components for assessing the risk for CAD much attention has been paid. The present study aimed to assess the relationship between ear characteristics (transverse groove on the earlobe and hair growth on the ear) and the risk for CAD and its severity among Iranian patients.

**Materials and Methods:**

In this cross-sectional study, the study population consisted of 105 consecutive patients with suspected CAD undergoing coronary angiography. The severity of CAD was determined by the number of disease vessels as well as the presence of left main lesions assessed by coronary angiography. All patients were examined to evaluate the appearance of ear regarding the presence of transverse groove on the earlobe and hair growth on the ear.

**Results:**

Comparing cardiovascular parameters across the groups with and without transverse groove on the earlobe showed a higher rate of CAD as well as the higher number of involved coronary arteries than in the groups without transverse groove on the earlobe. Similarly, the presence of CAD and its higher severity were more revealed in patients with hair growth on the ear as compared to the group without this phenotype. According to multivariable logistic regression analysis and with the presence of baseline parameters, the presence of transverse groove on the earlobe and hair growth on the ear increased the risk for CAD by 2.4 and 4.4 fold, respectively.

**Conclusion:**

Along with classic cardiovascular risk factors, the role of growing hair on the ear and transverse groove on the ear to predict high risk for CAD should be considered.

## Introduction

Coronary artery disease (CAD) is one of the major leading cause of mortality and morbidity, and even the second cause of death in most countries [1]. It has been estimated that by 2030 about one-third of the population in developed countries died or become disabled due to the progression of cardiovascular disorders and thus most of the healthcare costs will be allocated to the control and treatment of these diseases [2,3]. Till now, several planned approaches have been presented to predict the likelihood of significant CAD in suspected patients, especially in those with potential risk factors. Most of these algorithms are based on both clinical findings and imaging evidence achieving by invasive or semi-invasive modalities such as coronary angiography, perfusion scanning, and computed tomographic angiography [4,5]. Also, some specific limitations of such techniques due to their invasive nature or requiring radiation or contrasts agents, appearing complications following application of these procedures are not unexpected [6,7]. In this regard, the attention is focused on the simple naturally body markers for rapid predicting CAD risk. The earlobe characteristics, a simple clinical sign first described by Frank in 1973 as a good variable for predicting the risk of CAD [8]. Since then, some researchers have attempted to discover the pathogenic basis for the close link between parameters of earlobe and risk for CAD. Some studies described the close relationship between earlobe crease and increased risk for metabolic syndrome [9]. Also, some studies suggested a link between inflammatory markers such as macrophage activity and maintaining ear lobe collagen and loss of elastic fibers [10,11]. However, the relationship between earlobe parameters and CAD remains uncertain with a meaningful relationship in studies and the lack of relation in another study [12]. More importantly, this association is highly affected by racial features and genetic tendencies. Hence, the present study aimed to assess the relationship between ear characteristics (transverse groove on the earlobe and hair growth on the ear) and the risk for CAD and its severity among Iranian population.

## Materials and Methods

###  Patients

 In this cross-sectional study, the population consisted of 105 consecutive patients with suspected CAD undergoing coronary angiography at Imam Reza and Shahid Modaresi hospitals in Tehran, Iran, during 2017-2018. All patients underwent coronary angiography according to the standard protocol. Definition of CAD was based on more than 50% reduction in luminal diameter of the major coronary artery, or one of its major branches.

###  Sample Size Calculation

According to the findings of previous studies, to reach the objectives of our study, the sample size was calculated to be 105 patients [13].

###  Inclusion and Exclusion Criteria

 The patients with external ear disease or deformation, ear injury history, skin disease affecting ears, previous diagnosis of CAD and/or ischemic stroke, myocardial infarction, cardiomyopathy, and chronic diseases (such as cancer), were excluded from this study.

###  Data Collection

 The severity of CAD was determined by the number of disease vessels as well as the presence of left main lesions. Baseline characteristics including gender, age, patients’ weight and height, classic cardiovascular risk factors (e.g., hypertension, diabetes mellitus, smoking, alcohol consumption, and opium use) were collected by reviewing the hospital recorded files and entered into the study checklist. Along with reviewing the hospital files, all patients were examined for assessing the appearance of ear regarding the presence of transverse groove on the earlobe and hair growth on the ear. The study endpoint was to determine the relationship between ear characteristics and the presence and severity of CAD.

###  Ethical Statements

 The study protocol was approved by the Ethics Committees at Iran University of Medical Sciences (approval number: Ir.SBMU.msp.rec.1396.651).

###  Statistical Analysis

 Descriptive analysis was used to describe the data, including mean ± standard deviation (SD) for quantitative variables and frequency (percentage) for categorical variables. Chi-square test, independent t-test, and Mann-Whitney U test were used for comparison of variables. To determine the value of the transverse groove on the earlobe and hair growth on the ear to predict the presence of CAD, the multivariable logistic regression analysis was employed. For the statistical analysis, the SPSS Statistics for Windows version 22.0 (IBM Corp. Released 2013, Armonk, New York, USA) was used. P-values<0.05 were considered statistically significant.

## Results

The mean age of participants was 58.45 ± 13.46 years (ranged 30-91 years), and the mean body mass index was 26.23 ± 2.66 kg/m^2^. Regarding occupational status, 59.0% had an army-dependent occupation status, 26.7% were employed, 13.3% were self-employed, and others were unemployed. Concerning classic cardiovascular risk factors, 40.0% were a current or former smoker, 20.0% were opium user, and 6.7% were alcohol user. Also, 24.7% and 39.0% have diabetes mellitus and hypertension, respectively. According to the coronary angiography reports, 19.0% of participants had normal coronary condition, while minimal CAD was revealed in 22.9%, single-vessel disease in 15.2%, two-vessel disease in 21.0%, three-vessel disease in 17.1%, and left main lesion in 4.8%. The mean left ventricular ejection was 44.55 ± 11.09% in which 23.8% had ejection fraction lower than or equal to 35%. Comparison of baseline variables between the patients with and without transverse groove on the earlobe (Table-1) showed that the former group was significantly older and more hypertensive that as compared to those without this phenotype. Also, as shown in Table-2, those patients with hair growth on the ear were older, used alcohol more, and also suffered more from hypertension as compared to patients without hair growth on the ear. Comparing cardiovascular parameters across the groups with and without transverse groove on the earlobe showed a higher rate of CAD as well as the higher number of involved coronary arteries than in the groups without transverse groove on the earlobe (Table-3). Similarly, the presence of CAD and its higher severity were more revealed in patients with hair growth on the ear as compared to the group without this phenotype (Table-4). According to multivariable logistic regression analysis (Table-5) and with the presence of baseline parameters, the presence of transverse groove on the earlobe and hair growth on the ear increased the risk for CAD by 2.4 and 4.4 fold, respectively.

## Discussion

The use of phenotypic parameters along with other noninvasive diagnostic modality can lead to early diagnosis of CAD and to prevent its life-threatening outcome. Recently, the application of head and face components for assessing the risk for CAD much attention has been paid [12,13]. Even, the use of these parameters in new risk cardiovascular scoring systems has been highly recommended [14]. In this regard, some indicators such as shape or size of facial components have been indicated to have special role in the suggested scoring systems [11,12,15]. In line with previous studies on the association between ear characteristics and the risk for CAD, we examined the association between two parameters of the ear appearance including transverse groove on the ear and hair growth on the ear and presence of CAD [15]. We show that two pointed indicators could potentially predict CAD risk in suspected patients and candidate for coronary angiography. More interestingly, the predicting role of these two parameters is completely independent to other baseline CAD traditional risk profile. Till now, several cardiovascular risks scoring systems have been introduced such as the Framingham Coronary Heart Disease Risk Score [16], American College of Cardiology/American Heart Association (ACC/AHA) Risk score [17], the PREDICT-CVD secondary prevention score [18], atherosclerotic cardiovascular disease (ASCVD) [19], and WHO risk charts [20]. However, none of these scoring systems include apparent parameters. Regarding our findings, it can be strongly recommended to add these parameters, especially two tested parameters in the present study to modify current scoring systems and achieve new systems with higher sensitivity and accuracy. Regarding the association between transverse groove on the ear and the risk for CAD, reviewing the literature led to no similar studies. In other words, our study is the first survey on this association. Some studied focused on the relation between earlobe crease and increased risk for CAD. As indicated by Iorgoveanu *et al*. in 2018 [12], a close relationship could be suggested between the presence of the diagonal earlobe crease and CAD. A study by Honma *et al*. [14] in 2017, showed a close correlation between diagonal earlobe creases and coronary artery calcification score. In study by Aizawa *et al*. [15], the association between earlobe crease and left min coronary lesion has also been indicated. Regarding the pathogenesis of the increased likelihood of CAD in those with earlobe crease, some suggestions have been considered. Earlobe crease or Frank’s sign was first described by Sanders T. Frank in 1973 [16], as a predictive dermatological finding of coronary artery disease. The detailed mechanisms of developing earlobe crease is uncertain, although it is speculated to be derived from loss or degeneration of elastic fibers by diminishing blood supply to the earlobes in a pathological study. Ziyrek *et al*. [21] showed an association between the presence of diagonal earlobe crease and increased carotid intima-media thickness and epicardial adipose tissue thickness that the latter indicator can own strongly predict the severity of coronary artery disease. It also seems that the role of earlobe crease in the prediction of CAD can be augmented by the presence of other CAD risk factors such as hypertension and diabetes [21]. As shown by Kamal in 2017 [13], the effect of hypertension and diabetes on the presence of diagonal earlobe crease is statistically significant. Concerning the association between the hair on the ear and risk for CAD, almost all studies focused on the relation between ear-canal hair and CAD risk. Thus, the significant association between CAD and hair on the ear can be the novel aspect of our study. Although several various factors [22-25] can cause hair growth on the ear and may intervene (such as genetics, prostaglandins [E2 and F2α], growth hormone, insulin-like growth factor 1, transforming growth factor beta 1, tumor necrosis factor, basic fibroblast growth factor, thyroid hormones, glucocorticoids, estrogens, and androgens, etc.), based on the present study, growing hair on the ear (any point on auricle or earlobe) can powerfully predict the increased risk for CAD. The association between ear-canal hair and risk for CAD or acute myocardial infarction has also been pointed out in some previous studies [26,27]. In an early study by Verma *et al*. [23] in 1989, a significant difference was also observed between men with and without CAD in the presence of ear-canal hair. Similar findings were revealed by Wagner *et al*. [28]. In a study by Ali *et al*. in 2010 [29], 37.2% of CAD patients had hairy ear-canal, while the prevalence of other known risk factors for CAD such as hypertension or diabetes was lower. Also, they concluded that unknown risk factors for CAD such as hairy ear-canal might have a central role as compared to classic risk profile [29]. We also believed that the role of both ear-related indicators, including growing hair on the ear and transverse groove on the ear should not be ignored when modifying the scoring systems for predicting CAD and its severity.

## Conclusion

 Along with classic cardiovascular risk factors, the role of growing hair on the ear and transverse groove on the ear to predict high risk for CAD should be considered. Because of the pointed role for these indicators, considering such phenotypic components in modifying CAD risk scoring systems is quite logical.

## Acknowledgment

 Authors grateful to Parsian Teb Co. for their corrections pertaining to the English language.

## Conflict of Interest

 The authors declare no potential conflicts of interest.

**Table 1:** Comparing Baseline Parameters between the Groups with and without Groove on the Ear Lobe

**Variables**	**With groove** **(n=66)**	**Without groove** **(n=39)**	**P-value**
**Age, year**	61.88 ± 11.81	52.64 ± 14.23	0.001
**Body mass index, kg/m** ^2^	26.26 ± 2.68	26.17 ± 2.64	0.871
**Occupational status**			
Army-related	41 (62.1)	21 (53.8)	0.589
Employed	17 (25.8)	11 (28.2)	
Self-employed	7 (10.6)	7 (17.9)	
Unemployed	1 (1.5)	0 (0.0)	
**Smoking**	26 (39.4)	16 (41.0)	0.410
**Opium use**	15 (22.7)	6 (15.4)	0.363
**Alcohol consumption**	5 (7.6)	2 (5.1)	0.627
**Hypertension**	33 (50.0)	8 (20.5)	0.003
**Diabetes mellitus**	20 (30.3)	7 (17.9)	0.162

**Table 2:** Comparing Baseline Parameters between the Groups with and without Hair Growth on the Ear

**Variables**	**With ear hair** **(n=54)**	**Without ear hair** **(n=51)**	**P-value**
**Age, year**	61.96 ± 12.48	52.26 ±13.02	<0.001
**Body mass index, kg/m** ^2^	26.16 ± 2.76	26.34 ± 2.51	0.736
**Occupational status**			
Army-related	44 (65.7)	18 (47.4)	0.224
Employed	15 (22.4)	13 (34.2)	
Self-employed	7 (10.4)	7 (18.4)	
Unemployed	1 (1.5)	0 (0.0)	
**Smoking**	27 (40.3)	15 (39.5)	0.743
**Opium use**	13 (19.4)	8 (21.1)	0.839
**Alcohol consumption**	7 (10.4)	0 (0.0)	0.039
**Hypertension**	31 (46.3)	10 (26.3)	0.041
**Diabetes mellitus**	20 (29.9)	7 (18.4)	0.198

**Table 3:** Cardiovascular Status in the Groups with and without Groove on the Ear Lobe

**Variables**	**With baldness** **(n=54)**	**Without baldness** **(n=51)**	**P-value**
**Presence of CAD**	57 (86.4)	28 (71.8)	0.036
**Severity of CAD**			
None	9 (13.6)	11 (28.2)	0.007
Minimal CAD	11 (16.7)	13 (33.3)	
Single-vessel	8 (12.1)	8 (20.5)	
Two-vessel	19 (28.8)	3 (7.7)	
Three-vessel	15 (22.7)	3 (7.7)	
Left main lesion	4 (6.1)	1 (2.6)	
**Left ventricular ejection fraction**	41.50 ± 11.62	48.72 ± 10.68	0.002

**CAD: **Coronary Artery Disease

**Table 4:** Cardiovascular Status in the Groups with and without Hair Growth on the Ear

**Variables**	**With baldness** **(n=54)**	**Without baldness** **(n=51)**	**P-value**
**Presence of CAD**	60 (89.6)	25 (65.8)	0.003
**Severity of CAD**			
None	7 (10.4)	13 (34.2)	0.042
Minimal CAD	16 (23.9)	8 (21.1)	
Single-vessel	10 (14.9)	6 (15.8)	
Two-vessel	16 (23.9)	6 (15.8)	
Three-vessel	14 (20.9)	4 (10.5)	
Left main lesion	4 (6.0)	1 (2.6)	
**Left ventricular ejection fraction**	41.58 ± 11.73	49.17 ± 10.25	0.002

**Table 5:** Multivariable Logistic Regression Modeling to Assess the Value of Transverse Groove on the Earlobe and Hair Growth on the Ear in Predicting CAD

**Variable**	**B**	**S.E**	**Wald**	**OR**	**P-value**
Age	-0.061	0.032	3.664	0.940	0.056
Body mass index	-0.231	0.135	2.920	0.794	0.087
Smoking	0.249	0.691	0.130	1.283	0.718
Opium use	1.175	1.018	1.330	3.237	0.249
Alcohol use	0.247	1.322	0.035	1.281	0.852
Diabetes	1.878	0.883	4.521	6.543	0.033
Hypertension	2.376	0.940	6.389	10.756	0.011
Groove on the earlobe	-1.217	0.821	2.198	2.488	0.046
Hair on the ear	-1.249	0.766	2.654	4.457	0.038
Constant	-0.205	5.839	0.001	0.815	0.972
